# Incidence of COVID-19 in cancer patients in a teaching hospital faculty of medicine in Medan, Indonesia

**DOI:** 10.1016/j.ijregi.2023.03.008

**Published:** 2023-05-04

**Authors:** Dedy Hermansyah, Dede Kurniawan, Yolanda Rahayu, Batara Joseph, Fawzan Mohammad, Denny Rifsal Siregar, Emir Taris Pasaribu

**Affiliations:** a Division of Surgical Oncology, Department of Surgery, Faculty of Medicine, Universitas Sumatera Utara, Medan, Indonesia; b Department of Surgery, Faculty of Medicine, Universitas Sumatera Utara, Medan, Indonesia

**Keywords:** Cancer, COVID-19, Epidemiology, Indonesia

## Abstract

**Objective:**

The aim of this study was to determine the incidence of COVID-19 in cancer patients in the Teaching Hospital Faculty of Medicine, Universitas Sumatera Utara, Medan, Indonesia.

**Methods:**

This descriptive study was conducted at H. Adam Malik Hospital and USU General Hospital, Medan, from March 2020 to August 2021. The main inclusion criteria for the participants were COVID-19 and confirmed cancer. A total sampling technique was used.

**Results:**

Among the 3308 COVID-19 patients studied, the mean age was 44 years, ranging from 1 day to 92 years. Male participants were slightly more prevalent (51.8% vs 48.2%). Twenty-five of these patients (0.75%) had both COVID-19 and cancer, and thus met the inclusion criteria. The most common types of cancer were leukemia (20.0%), breast cancer (20.0%), ovarian cancer (16.0%), lymphoma (8.0%), and osteosarcoma (8.0%). The proportions of patients with mild, moderate, and severe degrees of COVID-19 where 48.0%, 24.0%, and 28.0%, respectively. Seven patients required intensive care, and five died.

**Conclusion:**

The incidence of COVID-19 in patients with cancer was relatively low. Nevertheless, almost a third of cancer patients with COVID-19 developed severe disease and had poor prognoses. Vaccination among cancer patients is important to protect them from poor outcomes if exposed to COVID-19.

## Highlights

• Patients with confirmed cancer plus COVID-19 will exhibit unfavorable outcomes.
• Most cancer patients with COVID-19 are in stage 4 of malignancy.
• Vaccination in cancer patients should not be delayed if the indication is met.

## Introduction

By November 18, 2021, global COVID-19 incidence had reached 254 256 432 cases in 251 countries. Indonesia was ranked 14th, with 4 251 945 cases. North Sumatra was among the top 11 Indonesian provinces for COVID-19 incidence, with 105 974 cases. Worldwide mortality was relatively high, at around 4% [1]. Men with comorbid hypertension and diabetes mellitus, as well as active smokers, are at risk of more frequent or susceptible occurrence of COVID-19. Patients with cancer and chronic liver disease are more susceptible to SARS-CoV-2 infection [2,3].

Cancer patients are placed under constant mental and physical stress, either due to the cancer itself or the long-term management with healing objectives in those patients, such as chemotherapy. Chemotherapy-based approaches have considerable and unfavorable side-effects, which in turn can leading to metabolic stress. The latter will induce a significant reduction in overall immune system response, hence placing cancer patients at risk for contracting infections, including several notable viral pathogens.

A previous review by Kamboj and Sepkowitz reported that cancer patients in epidemic (or pandemic) circumstances were significantly more exposed to viral infections due to impairment of host immunity [4,5]. Therefore, it is plausible to suggest that a similar phenomenon may be observed in the current COVID-19 pandemic. The first report on COVID-19 incidence in cancer patients was released on February 14, 2020, involving 18 Chinese patients with a history of cancer, who were diagnosed with COVID-19. In total, seven (39%) patients required treatment in the intensive care unit (ICU) and/or experienced mortality [3]. Worsening infections, poorer prognoses, a higher rate of ICU admission, a need for mechanical ventilation, and higher mortality were found in the cancer patient group when experiencing COVID-19 [6].

Another study in a Wuhan hospital found that cancer patients at the centre of the virus epidemic were at higher risk for contracting SARS-CoV-2 (OR = 2.31, 95% CI = 1.89–3.02) when compared with patients without cancer. However, fewer than half of those infected with COVID-19 were receiving active treatment for their cancer, and therefore immunosuppression could only partially explain the poorer prognoses following confirmation of COVID-19 [7]. A prospective analysis by Vuagnat et al. investigated the co-occurrence of COVID-19 in 76 breast cancer patients, with 77.6% of the participants confirmed as positive for SARS-CoV-2 infection as a result of radiological screening and routine preoperative examinations. The results showed that COVID-19 mortality in the study population was more dependent on associated comorbidities than on side effects associated with cancer treatment [8].

Therefore, a combination of awareness, observation, and early recognition regarding the incidence of COVID-19 in hospitalized cancer patients should be applied in every center, because the co-occurrence of both cancer and COVID-19 tends to place the patients at particular risk, according to several studies. This is especially the case in Indonesia, with its high population density and considerable prevalence of cancer patients. The aim of this study was therefore to assess the incidence of COVID-19 in cancer patients in the Teaching Hospital Faculty of Medicine, Universitas Sumatera Utara (H. Adam Malik Hospital and USU General Hospital, Medan).

## Methods

The included population for this observational descriptive study comprised cancer patients treated at H. Adam Malik Hospital and USU General Hospital, Medan, Indonesia from March 2020 to June 2021. A total sampling technique was applied in a cross-sectional analysis, using the centers’ medical records to collate data for all eligible and reliable participants who met the inclusion criteria. Ethical clearance for this study was granted by the ethical committees of both centers.

The inclusion criteria for the study were: inpatient and outpatient COVID-19 cases who had been diagnosed using a PCR-swab test; no previous history of contracting SARS-CoV-2; and a diagnosis of malignancy or cancer, as confirmed by histopathological analysis, with or without ongoing chemotherapy, radiotherapy, hormonal therapy, and surgical history at both centres. Accordingly, patients without or currently awaiting results of a PCR-swab COVID-19 test, suspected COVID-19 cases according to CT scan, and patients with pulmonary TB or pneumonia were excluded. The obtained data and patient information — all included in this report — were tabulated using SPSS 24.0 (Table 1).

**Table 1:** Demographic and clinical features of the participants.

Variables	Frequency (%)
**Gender**	
Female	1594 (48.2)
Male	1714 (51.8)
**COVID-19 incidence with cancer**	
Haji Adam Malik Hospital	24 (96.0)
USU General Hospital	1 (4.0)
**AJCC 8th edition cancer staging**	
Stage 1	0 (0)
Stage 2	1 (4.0)
Stage 3	7 (28.0)
Stage 4	12 (48.0)
Standard risk (leukemia)	5 (20.0)
**Cancer type**	
Lymphoma	2 (8.0)
Cervical	1 (4.0)
Endometrial	1 (4.0)
Leukemia	5 (20.0)
Osteosarcoma	2 (8.0)
Vulval	1 (4.0)
Ovarian	4 (16.0)
Renal	1 (4.0)
Urethral	1 (4.0)
Skin	1 (4.0)
Rectal	1 (4.0)
Breast	5 (20.0)
**Histopathology**	
Small cell NHL	1 (4.0)
Lymphoblastic leukemia	5 (20.0)
Non-keratinizing SCC	1 (4.0)
Keratinizing SCC	1 (4.0)
Osteosarcoma	2 (8.0)
Predominant Hodgkin-lymphoma	1 (4.0)
Low-grade adenocarcinoma	4 (16.0)
Mucinous carcinoma	1 (4.0)
Clear-cell carcinoma	2 (8.0)
Malignant melanoma	1 (4.0)
Well-differentiated adenocarcinoma recti	1 (4.0)
Invasive ductal carcinoma	4 (16.0)
Invasive lobular carcinoma	1 (4.0)
**COVID-19 severity**	
Mild	12 (48.0)
Moderate	6 (24.0)
Severe	7 (28.0)
**ICU and ventilator needs**	
Need for ICU care and ventilator	7 (28.0)
No need for ICU care and ventilator	18 (72.0)
**Patient outcome**	
Recovered	20 (80.0)
Deceased	5 (20.0)

## Results

This study initially encompassed 3308 COVID-19 patients diagnosed with COVID-19 using a PCR-swab test, of whom 2909 patients were treated at H. Adam Malik Hospital, while the rest were managed in USU General Hospital. The demographic and clinical descriptions of patients are shown in Table 1. The mean age of the initially identified COVID-19 patients was 44 years, ranging from 1 day to 92 years, while the mean age of COVID-19 patients with cancer was 52 years. Of the 3308 COVID-19 patients, 1714 were male and 1594 were female. In total, 25 patients (0.75%) had COVID-19 and cancer incidence. Of these, 24 patients were managed in H. Adam Malik Hospital and one patient in USU General Hospital.

According to AJCC (8th edition) staging, one patient had stage 2 cancer, seven patients had stage 3 cancer, and 12 patients had stage 4 cancer; the other five patients were being treated for standard-risk leukemia. With regard to cancer classification, several types were identified: breast cancer and leukemia (five cases each); ovarian cancer (four cases); lymphoma and osteosarcoma (two cases each); cervical, vulval, renal, urethral, skin, endometrial, and rectal cancer (one case each). Histopathology was distributed as follows: lymphoblastic leukemia (five cases); low-grade adenocarcinoma and invasive ductal carcinoma (four cases each); osteosarcoma and clear cell carcinoma (two cases each); non-keratinizing SCC, small cell NHL, keratinizing SCC, predominant Hodgkin-lymphoma, mucinous carcinoma, malignant melanoma, well-differentiated adenocarcinoma recti, and invasive lobular carcinoma (one case each).

COVID-19 can be graded, according to its sign and symptoms, as mild, moderate, or severe. In this study, 12 of the included patients were classed as mild cases, six as moderate, and seven as severe. ICU care, with the use of a ventilator, is usually required in patients with severe-to-critical COVID-19 symptoms. In this study, all seven patients with severe symptoms were treated in the ICU using a ventilator. Of the 25 included patients, five died and the remaining 20 recovered and were referred for outpatient treatment, giving a mortality rate of 20.0%.

## Discussion

The predisposition of cancer patients to severe COVID-19, or whether the patients with weakened immune systems, such as cancer patients, are susceptible to infection, remains unclear. It is likely that patients undergoing active chemotherapy or radiation therapy, especially those with blood malignancies, are at risk of developing severe COVID-19 and therefore have a worse prognosis [3].

With many health centres around the world taking precautions during the COVID-19 pandemic, there have been concerns over delays in cancer treatment, which have been associated with higher mortality from breast cancer and higher overall mortality [9]. Timely treatment throughout the course of breast cancer treatment is essential for optimal results. Most cancer centers discontinued non-emergency postsurgical monitoring, although policies varied across Asia, Europe, and the USA. A retrospective clinical study of the first cases of COVID-19 transmission showed that 41.3% of these occurred in hospitals. Most hospitals subsequently postponed planned operations, but this was unlikely to have affected the vast majority of cancer patients. Cancer management guidelines remained limited during the COVID-19 outbreak [10].

Another study in China concluded that the proportion of patients with a history of cancer was higher in the COVID-19 group than in the non-COVID-19 population, suggesting that patients with cancer are more likely to develop COVID-19. They found that out of 1590 COVID-19 patients in 575 hospitals in 31 provincial areas, 18 had a history of cancer. Treatment status was known in 16 of the 18 patients. In the previous month, four of the 16 patients had undergone surgery or chemotherapy, and another 12 (75%) had survived cancer, received routine care, and had not undergone immunosuppressive therapy. They concluded that COVID-19 infection in the 12 cancer survivors was related to their cancer history [11].

Of the 3308 COVID-19 patients initially included in our study, 25 were found to have cancer. These patients had an average age of 52 years. In a similar study conducted by Liang et al., the average age of these patients was significantly higher, at 63.1 years, suggesting that older age may be associated with worse COVID-19 outcomes. They observed patients with cancer to have a higher risk for severe events compared with those without cancer (7 [39%] of 18 patients vs 124 [8%] of 1572 patients; *p* = 0.0003). In addition, patients who had undergone chemotherapy or surgery in the previous month had a higher risk (3 [75%] of 4 patients) of severe clinical events than those who had not (6 [43%] of 14 patients) after adjusting for other risk factors, such as age, smoking history, and other comorbidities [3].

Our study captured 1714 male patients and 1594 female patients, of whom only 25 were included in the final analysis. Compared with men, women tend to experience COVID-19 more frequently. In contrary, previous research showed that compared with men, woman tend to experience COVID-19 more frequently. Toll-like receptors (TLR) and other immune-related genes present on the X chromosome are involved in the detection of single-stranded RNA viruses such as SARS-CoV-2. Innate immune cells are more active in women, making phagocytosis and the elimination of infected cells more effective. Estrogen is known to increase alpha-estrogen receptors in cytotoxic T lymphocytes, increasing interferon production. Thus, the virus elicits a stronger cytotoxic immune response in women, possibly contributing to a milder COVID-19 sequence [12].

Several studies have shown that the prevalence of COVID-19 in males is higher than that in females. One study demonstrated that SARS-CoV-2 infection tends to be found in older people of male gender, and is generally associated with comorbid diseases that can lead to fatal respiratory conditions, such as ARDS. Of the 99 COVID-19 patients in the study, 67 were men and 32 were women. However, in another study, a 1:1 male:female ratio was obtained from 140 COVID-19 patients with an average age of 57 years [2].

A 2020 study of COVID-19 patients identified the following cancer types: lung (seven cases, 25%), esophageal (four cases, 14.3%), breast (three cases, 10.7%), laryngocarcinoma, liver, and prostate (two cases each, 7.1%), cervical, gastric, colon, rectal, endometrial, ovarian, and testicular (1 case each, 3.6%) [2].

An investigation of 52 patients with concurrent COVID-19 and cancer reported that severe disease was associated with the following factors: use of immunosuppressants, such as steroids (*p* = 0.001); hemodynamic decompensation patterns, such as severe acute respiratory syndrome (*p* < 0.001), myocardial injury (*p* = 0.04), and shock (*p* = 0.02), as well as serum markers of inflammation, such as lymphopenia and elevated levels of IL-6, D-dimer, C-reactive protein (CRP), pro-calcitonin, and lactate dehydrogenase (LHD) (*p* < 0.05).

Two studies in Europe confirmed that advanced age predicts higher mortality. Another study, involving a series of 25 cancer patients with COVID-19 in northern Italy, found that female gender (*p* = 0.04), multiple primary tumor locations (e.g. genitourinary and hematologic) (*p* = 0.02), and elevated CRP (*p* = 0.047) were associated with higher mortality [13]. Research in Paris included 59 patients with breast cancer and found that high blood pressure (*p* < 0.05) was also associated with severe infection. There was no relationship between the extent of lung lesions caused by COVID-19 and the degree of previous radiation therapy or pulmonary sequelae [8].

Mehta et al., in a study of 218 New York cancer patients with COVID-19, reported that factors associated with an increased risk of death were older age (> 65 years; *p* = 0.0006), heart disease (*p* = 0.012), coexisting chronic lung disease (*p* = 0.0003), ICU requirement (*p* < 0.001), and elevated serum inflammatory markers, such as D-dimer (*p* = 0.002), DHL (*p* = 0.01), and lactate (*p* = 0.001) [14].

From the above, concerns that cancer patients might be more at risk for COVID-19 infection appear to be unfounded. Many factors can increase the risk of COVID-19 infection, but the immunosuppressed status of cancer patients appears not to render them more vulnerable to COVID-19 infection, or indeed severe symptoms. Cancer patients who have undergone chemotherapy, immunotherapy, or radiotherapy do not appear to be associated with an increased risk of COVID-19 infection [14].

Our study presented descriptive data for cancer patients who were simultaneously infected with SARS-CoV-2 between March 2020 and June 2021. The aim was to increase understanding of the influence of cancer and COVID-19 on the immune system, and thus the effects on overall outcomes, since the correlation between both variables had not been clearly defined to date. This also relates to the suggestion that that COVID-19 may weaken the immune response to cancer, thus amplifying the associated morbidity or even mortality. However, one limitation of this study was a lack of any control group, to compare the influence of COVID-19 in both cancerous and non-cancer patients, which would have changed the design of this study to an analysis investigation. Further larger-scale analyses are required to ascertain the mutual influences of COVID-19 and cancer, in order to guide future clinical guidelines.

## Conclusion

The relatively poor prognosis for COVID-19 infection among cancer patients in some centres should raise awareness regarding the possible effects on the patients’ overall immunity and susceptibility to more severe forms of infection. The implication is that vaccination programs among cancer patients should be prioritized to prevent further exposure to SARS-CoV-2 during in-hospital management.

## CRediT authorship contribution statement

**Dedy Hermansyah:** Conceptualization, Data curation, Formal analysis, Investigation, Methodology, Project administration, Resources, Supervision, Validation, Visualization, Writing – original draft, Writing – review & editing. **Dede Kurniawan:** Conceptualization, Data curation, Formal analysis, Investigation, Methodology, Resources, Software, Validation, Writing – original draft, Writing – review & editing. **Yolanda Rahayu:** Conceptualization, Data curation, Formal analysis, Investigation, Resources, Software, Writing – original draft, Writing – review & editing. **Batara Joseph:** Conceptualization, Data curation, Investigation, Resources, Software, Visualization, Writing – original draft, Writing – review & editing. **Fawzan Mohammad:** Conceptualization, Data curation, Investigation, Resources, Software, Visualization, Writing – original draft, Writing – review & editing. **Denny Rifsal Siregar:** Project administration, Resources, Supervision, Validation, Writing – review & editing. **Emir Taris Pasaribu:** Project administration, Resources, Supervision, Validation, Writing – review & editing.

## Conflicts of interest

None declared.
